# Health capability of family caregivers: how different factors interrelate and their respective contributions using a Bayesian approach

**DOI:** 10.1186/s12889-016-3027-8

**Published:** 2016-04-28

**Authors:** Barbara Bucki, Elisabeth Spitz, Anne-Marie Etienne, Etienne Le Bihan, Michèle Baumann

**Affiliations:** University of Luxembourg, Research Unit INSIDE, Institute of Health & Behaviour, Maison des Sciences Sociales, Campus Belval, L-4633 Esch-sur-Alzette, Luxembourg; University of Lorraine, University Paris Descartes, APEMAC EA4360, Campus du Saulcy, 57006 Metz, France; Unité de psychologie de la santé, Faculté de Psychologie, Logopédie et Sciences de l’Education, University of Liège, 4000 Liège, Belgium

**Keywords:** Health capability of family caregivers, Bayesian approach, Fatigue, Stroke, Feeling abandoned, Material conditions, Health inequalities

## Abstract

**Background:**

The lifestyles of family caregivers pose risks to their physical, mental and social health. The capability to stay healthy may be protective in the context of poor socioeconomic conditions and risk behaviours, but the interrelations between its aspects and their respective influences remain unclear. The aim of this study was to evaluate the interrelations between the factors comprising health capability of family caregivers (HCFC) and the respective contributions of its components.

**Methods:**

All stroke patients admitted to all hospitals in Luxembourg were identified by the ‘*Inspection Générale de la Sécurité Sociale*’ using the national database system for care expenditure reimbursement, and asked to designate the main person caring for them. Sixty-two caregivers (mean age 59.3 years; 40 women and 22 men) responded face to face, to a questionnaire including 20 items measuring eight aspects of health capability (physical functioning, psychological functioning, lifestyle value, self-efficacy towards the use of health services, family support, social capital, material conditions/sense of security, and satisfaction with the interactions with health services). Using a Bayesian approach, significance values were estimated by comparing the test values to the posterior distribution of the parameters. Structural equation modelling with standard deviations was applied.

**Results:**

Female family caregivers had lower scores than men in physical and psychological functioning. Family caregivers with the lowest incomes had the least lifestyle value, social capital and material conditions/security. Self-efficacy towards health services increased with age. The material conditions/sense of security factor was positively correlated with almost all the others. The items that impacted health capability factors the most were - for physical functioning – fatigue, and - for family support - feeling abandoned by the family.

**Conclusions:**

During the chronic phase, relationships between risk behaviours can help guide social and health decision-makers to determine their priorities in improving the lives of family caregivers. Enhancing health capability involves implementing programs that relieve family caregivers physically, and foster family networking around the person being cared for. Special attention should also be paid to the socially disadvantaged in order to fight inequalities in health capability.

**Electronic supplementary material:**

The online version of this article (doi:10.1186/s12889-016-3027-8) contains supplementary material, which is available to authorized users.

## Background

Along with demographic changes (increased life expectancy) and economic recession (increasing social inequalities and health inequalities), the increasing prevalence of chronic disorders such as cerebrovascular disease [1] produces new concerns that call for intergenerational solidarity. The use of public health services is growing, but at the same time, the diminution of public finances has contributed to shorter hospital stays [2]. The introduction of new modes of care (e.g. day hospitals) and the implementation of interventions in order to delay institutionalization of vulnerable individuals [3] are only few examples showing that part of the responsibility for ongoing care has been placed on families.

Caregiving is based on an emotional relationship, and family caregivers can take pleasure from caregiving-related activities [4]. From their point of view, the disease allows them to discover their strengths, and to strengthen ties with the person being cared for [5, 6]. However, the physical activities and responsibilities required of family caregivers are demanding. As demonstrated by two meta-analyses, compared to non-caregivers, family caregivers are at increased risk of developing both physical [7], and mental health problems [8]. As their social interactions decline over time, they have less and less opportunity to receive support, both instrumentally and emotionally, which makes them vulnerable [9]. Interference with employment may also intensify the caregiving burden [10]. As the lifestyles of caregivers pose health risks [11, 12], maintaining their well-being has become a topical challenge for public health services [13]. In that context, having a high health capability is protective. In a general sense, capability is what a person can do or be according to the choices that are actually available [14, 15]. Among family caregivers, relevant aspects of capability include the capacity to get on with the person being cared for, to obtain institutional support, to receive informal support, to have activities outside caregiving, to feel in control of the caring, and to gain fulfilment from it [16]; the most satisfied people being those who can – if they wish - participate in activities outside caregiving and get on with their relatives [17]. Situated at the intersection between personal, social, environmental and political factors, as well as the quality of public health and of the health care system, the Health Capability paradigm aims at recognizing all the factors that influence the capacity to achieve optimal health [18]. A recent study based on this paradigm highlighted eight factors of health capability of family caregivers [15]: psychological functioning, physical functioning, self-efficacy toward health services, lifestyle value, family support, social capital, security/material conditions, interactions with health services.

Among family caregivers, all the domains of quality of life - physical, psychological, environment, and social relationships - as measured by the Whoqol-bref [19] are positively associated with life satisfaction [20]. Caregivers who lack family support, have their schedules disrupted and financial problems due to caregiving – as measured by the Caregiver Reaction Assessment [21] – have a lower physical quality of life [22]. Regarding social and health services, the most unsatisfying aspects concern information about the role of caregiver, help applying for benefits and services, and modification of services in response to change [23], as measured by the Satisfaction with Community Services questionnaire (CSCS) [24].

The literature clarifies the relationships between factors in terms of simple comparisons. For example, caregivers with high levels of physical functioning tend to function better psychologically [25, 26]. Caregivers who hold their role in high esteem have a higher overall quality of life than those who do not [27]. Physical and psychological functioning are both associated with a sense of self-efficacy regarding the use of community services [28] but are also impeded by financial constraints [29, 30]. Although family support fosters physical functioning [31] and is protective against depression [27], a lack of family support did not seem to impede general psychological functioning [25]. The use of health and social services also helps with adaptation to the caregiving role as family caregivers who use in-home services early in their caregiving career tend to delay the institutionalization of their relatives with dementia [26]. After a relative’s stroke, support from community services did not promote a better quality of life in family caregivers, nor diminish the perception of daily tensions [32, 33]. Inequalities in health and mortality between groups with different socioeconomic profiles has been extensively documented [34]. Similarly, socioeconomic conditions of family caregivers may engender different profiles of caregiving.

These results give partial clues to the complex relationships between factors affecting the health capability of family caregivers. The capability to be healthy is certainly protective in a context of poor socioeconomic conditions and risk behaviours. However, how factors interrelate remains unclear. Identifying the relationships between aspects of health capability, and, within them, the components that contribute most, will help guide health decision-makers to determine their priorities when helping family caregivers.

The aim of this study was to evaluate the relations between each of eight factors of health capability of family caregivers, and the contribution of each item to each factor.

## Methods

### Study design, sample and recruitment

All victims of a stroke in Luxembourg were identified by the ‘*Inspection Générale de la Sécurité Sociale*’ using the national database for care expenditure.

### Inclusion criteria

- Living in Luxembourg at the time of cerebrovascular disease onset;
- Having been hospitalised in Luxembourg over a period of 18 months;
- Having a clinically diagnosed stroke (hospital discharge code based on the International statistical classification of diseases and related health);
- Living in Luxembourg and not in an institution two years after the stroke onset;
- Understanding one of the four following languages: Luxembourgish, Portuguese, French, German.

Of the 374 patients concerned, 102 agreed to be contacted by telephone to arrange an appointment during which they designated, if necessary, their main caregiver as ‘the person who helps [them] most since the stroke’. Caregivers so designated were invited to participate in the survey. From the 76 households that participated, 62 caregivers were interviewed at their homes, face to face by a researcher.

### Ethical aspects

The study protocol was approved by the National Committee of Research Ethics (NCRE) and notified to the Committee for Data Protection of Luxembourg. An informational letter and a request for written informed consent were sent to 374 patients to: obtain their agreement to consult their hospitalization and rehabilitation records; explain the aims of the national sur vey; and request authorisation for a researcher to visit their home. Clinical diagnosis of cerebrovascular disease was confirmed by the medical investigator. Written informed consents of the main caregivers were obtained at the first visit.

### Data collected

Two researchers, one per interview, conducted the structured interviews supported by a questionnaire. The Health Capability of Family Caregiver (HCFC-8 factors) instrument is composed of 20 items stemming from three questionnaires: the Whoqol-bref quality of life [19], the Caregiver Reaction Assessment (CRA) [21], and the Carer Satisfaction with Community Services (CSCS) [24]. Following guidelines for shortening composite measurement scales, the number of items was reduced in order to maintain both content validity and psychometric properties [35] (see Additional file 1):

- *psychological functioning* (3 items; Cronbach α = 0.71): self-esteem, body image, and negative affects (reversed); from the Whoqol-bref “psychological” factor;
- *physical functioning* (2 items; α = 0.77): feeling tired (reversed) and health got worse (reversed); from the CRA “Impact on health”.
- *self-efficacy towards health services* (2 items; α = 0.80): confidence to know who to contact and ability to get information; from the CSCS;
- *lifestyle value* (3 items; α = 0.76): want to care, enjoy caring, and caring makes one feel good; from the CRA “caregiver esteem”;
- *family support* (3 items; α = 0.71): difficulty getting help (reversed), feeling abandoned (reversed) and family works together; from the CRA “lack of family support”;
- *social capital* (3 items; α = 0.70): personal relationships, sexual activity and social support; from the Whoqol-bref “interpersonal relationships”;
- *material conditions/security*: (2 items; α = 0.70): financial resources, freedom and physical safety and security; from the Whoqol-bref “environment”;
- *quality of information and healthcare services* (2 items; α = 0.82): help received and availability of information; from the CSCS questionnaire.

In line with the original questionnaires, each item was measured with a 5-point Likert scale. The higher the score, the better the health capability of family caregivers.

#### Socioeconomic characteristics

The following socio-demographic characteristics were collected: age, sex, type of relationship with the patients (partners vs. other), educational level (under 12th grade; 12th grade and above), occupation at the time of the stroke onset (never employed; manual worker; employee/intermediate professional/technician; farmer; manager/professional), current occupational status (working; retired; unemployed), income (cut-off point of 36,000€; representing three times the minimum wage in 2008).

### Translation of the instruments

As Luxembourg is multilingual and culturally diverse (more than 170 different nationalities), our questionnaires were available in four languages: Luxembourgish, Portuguese, French and German. Most of the instruments were already available in French or English. The German, Portuguese and Luxembourgish versions were translated and back-translated, then proofread by native-speaking professional translators. As Luxembourg does not have academic medical facilities, all neurologists were trained elsewhere in Europe. The Luxembourg Society of Neurologists includes specialists who speak many languages and are culturally diverse. They collaborated in supervising the conception of all documents, the questionnaire for the patients and the caregivers, and their translation.

### Statistical analyses

The Bayesian approach uses the algorithms of Monte Carlo Markov Chain (MCMC) [36]. It requires no asymptotic approximation and is also suitable for modest sized samples. In addition, this approach can treat missing data appropriately thanks to the technique of data augmentation. Thus, it is particularly suited to the implementation of our complex methodology.

Our approach was to associate each item in an ordered response (which corresponds to all of our items) to a continuous latent variable whose distribution is assumed to be normal [37]. Each latent variable comprises a series of thresholds so that a value between two thresholds determines the category of the response to the corresponding item [38]. The latent variables associated with items then play the role of manifest variables in the classical description of structural equation models. These variables are called “manifest” latent variables (MLV). This procedure eliminates an assumption that the item responses follow a continuous normal distribution (an assumption underlying the classical statistical approach), which can lead to false conclusions, especially when distributions are skewed as was the case in our sample. From this point on, in order to facilitate understanding of the results, the MLVs related to each item will simply be called “items”.

**Fig. 1:**
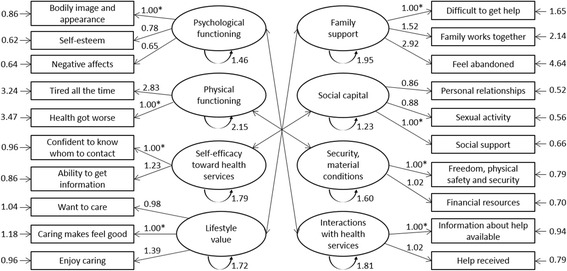
Factor loadings, standard deviation of the latent factors and items of HCFC

We then performed a confirmatory factor analysis. The MLVs were related to the latent factors as shown in Fig. 1. The latent factors were assumed to follow a multivariate normal distribution and were allowed to correlate with each other.

The model was implemented through OpenBUGS software [39]. The laws of prior distribution parameters were chosen so that they are non-informative: normal laws with zero expectation and variance 10^6^ for factor loadings, uniform distribution between 0 and 100 for the variances of the MLV and Wishart distribution expecting the identity matrix with 10° of freedom. Furthermore, thresholds for associating items to MLV were determined assuming that the latter had a standard normal distribution. They were estimated simultaneously with the other parameter of the model. The means of the MLV were set to 0 and the factor loading of the MLV was set at 1 for each latent factor, so as to ensure that each model parameter was identifiable. Significance values were estimated by comparing the test values to the posterior distribution of the parameters.

## Results

### Description of the sample

The 62 participating family caregivers (mean age 59.3 years) were 40 women and 22 men (Table 1). Two years after the stroke, about a quarter are aged over 67 years. They were mainly spouses of the stroke victims −51 couples- and 15 % were a child or someone close to the family. Mostly from the professional class of employees and technicians, more than half had an educational level of more than 12 years. Two years after the stroke of their relative, one third pursued a professional activity, while another third was retired and the last third did not work.

**Table 1:** Socio-demographic and health characteristics of the respondents

		Family caregivers
		*n* = 62
		%
Sex	Female	64.5
	Male	35.5
Relationship caregiver/stroke patient	Partner	85.0
	Child	10.0
	Other	5.0
Profession	Never worked	17.5
	Manual worker	14.0
	Employee, technician	49.1
	Executive, independent	19.3
Educational level	<12th grade	42.4
	≥12th grade	57.6
Occupational situation	Active	35.6
	Retired	33.9
	Inactive	30.5
Income	<36 000€/year	28.9
	≥36 000€/year	71.1
		M (s)
Age		59.3 (13.7)

### Associations between the socio-demographic characteristics and health capability scores of family caregivers

Women had lower scores than men in physical and psychological functioning. Family caregivers with an income of below 36 000€/year had lower scores for lifestyle value, social capital and material conditions/security. Perceived self-efficacy towards the use of health services increased with age (Table 2).

**Table 2:** Health capability factors according to socio-demographic characteristics of the family caregivers

	Psychological functioning [0;100]			Physical functioning [0;100]			Self-efficacy health services [0;100]			Lifestyle value [0;100]		
	m	s	*p*	m	s	*p*	m	s	*p*	m	s	*p*
Sex												
Female	68.9	20.5	0.007**	57.6	34.1	0.010*	66.8	29.5	0.671	76.5	20.7	0.240
Male	83.3	15.5		80.9	22.1		63.1	33.6		85.4	12.4	
Relationship with stroke patient												
Partner	74.0	20.4	0.845	69.9	31.1	0.171	67.3	30.8	0.278	80.9	16.4	0.125
Other	75.5	19.7		51.8	34.9		53.6	33.6		69.0	27.9	
Profession												
Never worked	70.4	21.2	0.783	56.2	34.7	0.288	79.7	29.1	0.406	79.2	28.9	0.443
Manual worker	68.7	30.1		50.0	31.3		51.6	33.7		67.7	16.9	
Employee, technician	76.2	16.5		69.9	34.5		62.5	33.3		82.4	14.1	
Farmer	83.3	23.6		100.0	0.0		56.2	44.2		83.3	0.0	
Executive, independent	70.4	23.6		78.1	23.9		72.2	18.5		82.3	15.7	
Educational level												
< 12th grade	68.3	24.7	0.091	59.7	34.7	0.219	65.1	32.8	0.968	75.8	21.2	0.155
≥12th grade	77.3	15.5		71.6	28.4		64.8	29.6		83.7	14.7	
Occupational situation												
Active	76.1	20.3	0.818	64.7	31.6	0.342	56.0	32.6	0.147	79.9	16.2	0.606
Retired	72.7	22.7		54.2	32.1		74.2	30.8		76.4	25.1	
Inactive	75.6	17.9		83.0	21.8		64.2	27.9		80.3	15.9	
Income												
< 36 000€/year	65.7	26.1	0.070	47.9	24.9	0.027*	58.3	35.1	0.397	70.8	24.5	0.039*
≥ 36 000€/year	78.4	18.2		73.4	33.6		67.7	31.3		84.8	14.1	
Age	Corr. coeff.		*p*	Corr. coeff.		*p*	Corr. coeff.		*p*	Corr. coeff.		*p*
	0.10		0.453	0.03		0.821	0.26		0.046*	0.17		0.251
	Family support [0;100]			Social capital [0;100]			Material conditions/Security [0;100]			Interactions with health services [0;100]		
	m	s	*p*	m	s	*p*	m	s	*p*	m	s	*p*
Sex												
Female	68.7	28.8	0.104	76.4	14.4	0.402	74.7	21.3	0.497	56.9	29.8	0.249
Male	82.3	15.5		79.6	13.1		78.7	22.6		65.8	19.5	
Relationship with stroke patient												
Partner	74.1	24.6	0.712	78.5	12.8	0.030*	75.5	22.3	0.804	62.0	27.0	0.157
Other	70.2	30.4		67.2	16.7		73.4	17.0		46.4	23.6	
Profession												
Never worked	74.0	34.6	0.251	78.3	20.0	0.925	73.7	16.1	0.112	77.8	23.2	0.302
Manual worker	56.2	25.5		76.0	10.4		57.8	32.7		54.7	26.7	
Employee, technician	79.9	22.4		77.1	13.3		81.2	19.7		59.1	25.1	
Farmer	91.7	0.0		70.8	5.9		75.0	17.7		75.0	0.0	
Executive, independent	76.0	15.7		80.1	12.5		73.6	17.1		56.9	28.0	
Educational level												
< 12th grade	74.2	23.7	0.884	75.2	11.2	0.372	72.0	20.2	0.271	59.1	27.3	0.840
≥12th grade	73.1	27.7		78.4	15.3		78.3	22.5		60.6	26.9	
Occupational situation												
Active	73.0	24.4	0.790	74.6	12.4	0.722	74.5	25.7	0.668	52.7	25.8	0.458
Inactive	74.3	28.3		78.9	17.8		78.9	16.3		63.5	29.9	
Retired	76.5	17.4		80.6	11.7		76.7	15.6		63.4	27.5	
Income												
< 36 000€/year	64.6	28.5	0.170	67.9	12.3	0.008**	54.8	27.7	0.000***	55.2	32.6	0.246
≥ 36 000€/year	77.9	25.7		80.2	13.8		84.8	13.0		65.5	22.1	
Age	Corr. coeff.		*p*	Corr. coeff.		*p*	Corr. coeff.		*p*	Corr. coeff.		*p*
	−0.00		0.987	0.07		0.585	0.18		0.173	0.22		0.108

### Correlations between health capability factors

All the health capability factors were positively correlated with each other and half the correlations were significant (Table 3).

**Table 3:** Correlation matrix between health capability factors

	Psycho health	Physical health	Self-efficacy	Lifestyle value	Family support	Social capital	Material, security
Physical health	0.350	1					
Self-efficacy towards health services	0.246	0.422	1				
Lifestyle value	0.319	0.425*	0.435*	1			
Family support	0.310	0.571**	0.357	0.604***	1		
Social capital	0.322	0.535**	0.333	0.326	0.418*	1	
Material conditions, security	0.589***	0.473*	0.392*	0.493*	0.429*	0.362	1
Interactions with healthcare services	0.327	0.406	0.647**	0.332	0.341	0.328	0.407*

Significance levels: *p* < 0.05*; *p* < 0.01**; *p* < 0.001***

The ‘material conditions/sense of security’ factor was correlated with all other factors except social capital. Physical functioning was strongly correlated with social capital and family support, and to a lesser extent with lifestyle value. Self-efficacy towards health services was particularly associated with the quality of interactions with health services and lifestyle value. Lifestyle value was also linked with self-efficacy, family support and physical functioning. Finally, psychological functioning was significantly correlated with no other factor except material conditions/sense of security.

### Contribution of items to health capability factors

Factor loadings and standard deviation of the latent factors and items are presented in Fig. 1. Physical functioning was mainly linked with the item regarding “feeling tired all the time” (factor loading- fl - 2.83 vs. 1.00 for “health got worse”). Family support was the most strongly represented by the item assessing “feeling abandoned” (factor loading 2.92). With slight differences compared to the other items composing the factors (±0.40), bodily image represented psychological functioning the most, and enjoying caring was the most important to lifestyle value (fl 1.39). Within security/material conditions and interactions with health services, all the items had similar importance (fl ranging from 1 to 1.02).

## Discussion

Conducted at the chronic phase, our study analysed the contributions of each risk factor and behaviour marker, and determined their global contributions to the health capability of family caregivers (HCFC). The HCFC instrument appeared to be an appropriate prototype with which to produce useful indicators to be considered in programs for family caregivers of stroke patients with disabilities. It will be useful for the evaluation of the efficiency of health intervention, as well as in clinical practice to determine the needs of family caregivers.

In our results, HCFC was mostly hindered by a combination of feeling tired and feeling abandoned by the family; representing, respectively, troubles with physical health and family support. These factors appeared to be interrelated. Previous studies have shown that fatigue can have deleterious effects on the immune system by placing people at cardiovascular risk and altering the management of their emotions [40, 41]. Family support has been documented in the literature. In particular, a lack of it is associated with the presence of depressive symptoms [27]. Our results also highlight the fact that social support from the family in caring tasks is associated with the least fatigue and deterioration in health. Similarly, we observed that physical health was also related to higher social capital, and higher lifestyle value. This finding reinforces the idea that health capability of family caregivers relates to the quality of their perceived social support, including by allowing them to be relieved from physical strain. Thus, there is a need to implement interventions that foster family networking around the patient.

Another finding is that the material conditions/sense of security factor was associated with all the other factors except social capital, whereas psychological functioning appeared more independent. Regarding the socio-economic profile of our sample, we found that family caregivers with the lowest incomes had the lowest scores of health capability factors. In other words, socioeconomically disadvantaged family caregivers are those with least levels of health capability. Contextualising our findings poses a challenge for a number of reasons; in particular, the economic situation (as regards Luxembourg’s gross domestic product per inhabitant), and the fact that Luxembourg is one of the smallest European countries (2 600 km^2^; 524 853 inhabitants in January 2012) with short distances between individuals and health facilities. Care is thus geographically accessible for the whole population. The socio-demographic characteristics of the study sample (71.1 % had an income of 36 000€ or more) also suggest that social and medical services were probably more easily available for them than other populations. Difficulties associated with maintaining inner-city medical practices [42] and community-care provision vary substantially according to location and income. These factors also influence domiciliary care delivery: distribution of resources at local levels; financial constraints; and the application of eligibility criteria in providing medical and community services [43].

Social inequalities, systematic differences in health between different socioeconomic groups within a society, exist [44]. Despite constant efforts over several decades by European countries to reduce these gaps [45], health inequalities persist [46]. This observation suggests there is a need to re-think the way future measures to counter health inequalities among family caregivers are conceived. It will be necessary to investigate the priority of their needs; whether socially disadvantaged caregivers need personal skills, strengthened communities, improved living and working conditions and access to essential services, or better healthy macro-policies [47]. Overall, we suggest that strengthening caregivers’ networks around the patient can be beneficial, but further studies will help determine if the needs of caregivers who are socially disadvantaged differ from those of caregivers who are not.

Surprisingly, the relation between psychological and physical functioning has not been verified. This result is not in line with the literature which shows instead a strong link between these factors [25, 26]. However, this finding can be interpreted by taking into account the themes covered by the questionnaire. Psychological functioning is measured by relatively stable traits which develop during life, such as self-esteem or body image. In contrast, the items that measure physical functioning relate to states associated with the effects of stroke and caregiving. Thus, this absence of relation can mean that the physical strain caused by caregiving did not tarnish the image people have of themselves as family caregivers.

On the other hand, satisfaction with interactions with health and social services was not related to physical and psychological functioning. Interpretation of this result would require knowledge of how satisfaction changes according to the frequency of use. In this study, our sample brings together a range of family caregivers varying from those who reported no sequelae, to those who experienced a variety of effects. Patients with most sequelae are likely to have the most repercussions of stroke on their health. Thus, they and their family caregivers are assumed to be the group most likely to have used health and social services. Therefore, in our sample, the lack of an association between satisfaction with health services and physical or psychological functioning may reflect the fact that availability and/or use of health services allowed for maintenance of an average health status. In addition, in our sample, older family caregivers had a higher self-efficacy towards the use of health services. That result means that there is a lifelong learning about the skills to be acquired. More practically, it highlights the potential beneficial effects on health capability of informing family caregivers about the available opportunities from the very beginning of the chronic disease of their relatives.

These findings need to be interpreted taking into account the socio-demographic profile of our sample. The literature shows that physical and psychological functioning is lower in women than in men. Yet in our study, two thirds of the family caregivers were women. Caring differs according to gender [48, 49] – women seemingly feel more tired than men and are more likely to put aside their own activities in favour of caring [50]. Moreover, it is known that women live longer than men, which means that they spend more time than men with impaired health [51]. Further studies with larger samples and involving more men will determine how health capability differs between male and female family caregivers.

### Strengths and limitations

Studying the health capability of family caregivers at the chronic phase of their relatives’ disease is an opportunity to provide valuable information on patient-caregiver monitoring over time. In addition, after two years, stroke patients and their family caregivers may have adapted to their new situation, reorganized their daily lives, and become accustomed to caregiving [52]. Using the Bayesian approach overcame the constraints of the small sample size. Contrary to the so-called classical statistical approach, it relies on effective data, so that the obtained model is likely to reflect reality while keeping statistical rigor. The consistency in the meaning of the results encourages the reproduction of this study with larger samples; which will allow for the model to be adjusted accordingly.

Assessing health capability requires having information on health status, but also on the actions taken to achieve that status, and to what extent these two first indicators are the result of a choice [18]. Our conceptual model composed of eight factors would benefit from being completed by markers that fall into the emotional sphere, which is central to caregiving [53].

### Practical implications

Current information and communication technologies (ICT) offer new insights into the promotion of health capability allow family caregivers to get information and develop skills while staying at home, but connected to others (using phone, videoconferences, telestroke) [54]. A research-action program using ICTs could be implemented to enhance health capability among family caregivers. It would initially identify the factors of health capability to be improved by determining the needs of family caregivers, based on the theoretical model and a qualitative analysis from interviews and focus groups. Second, corresponding ICT applications can be created by gathering the expertise of multisectoral partners from the research, medical, associative and private sectors (with the help of technology companies). Third, the efficacy of the health capability program will be tested with a case–control study, and its impact on the quality of life of the dyads will be evaluated. Bringing together bottom-up and top-down approaches, such a project develops primary prevention tailored to the needs of family caregivers, taking into account their environmental context. It thus promotes an innovative health-in-all approach.

## Conclusions

The HCFC instrument is composed of inter-related factors at the center of which lay ‘material conditions’, indicating that special actions should be directed to the socially disadvantaged. On the other hand, interventions that would have the most benefit have to relieve family caregivers from physical strain and foster family networking around the person being cared for; all of these in order to prevent any one particular person from bearing the burden of caregiving alone.

### Ethics approval and consent to participate

The study protocol was approved by the National Committee of Research Ethics (NCRE) and notified to the Committee for Data Protection of Luxembourg. An informational letter and a request for written informed consent were sent to patients to: obtain their agreement to consult their hospitalization and rehabilitation records; explain the aims of the national survey; and request authorisation for a researcher to visit their home. Clinical diagnosis of cerebrovascular disease was confirmed by the medical investigator. Written informed consents of the main caregivers were obtained at the first visit.

## Additional file

Additional file 1: Prototype of the Health Capability of Family Caregivers questionnaire (Bucki, 2014). (DOC 79 kb)

## Abbreviations

CRA: Caregiver Reaction Assessment
CSCS: Carer Satisfaction with community services
HCFC: health capability of family caregivers
MLV: “manifest” latent variable.
