# Supplementary material for: Health capability of family caregivers: how different factors interrelate and their respective contributions using a Bayesian approach
Source: BMC Public Health. 2016 Apr 28;16:364. doi: 10.1186/s12889-016-3027-8 (PMC4848818; doi:10.1186/s12889-016-3027-8)
Supplement: Additional file 1: — Prototype of the Health Capability of Family Caregivers questionnaire (Bucki, 2014). (DOC 79 kb) [file 12889_2016_3027_MOESM1_ESM.doc]

Appendix 1. Prototype of the Health Capability of Caregivers questionnaire

|  | Strongly agree | Agree | Neither agree nor disagree | Dis-agree | Strongly disagree |
| --- | --- | --- | --- | --- | --- |
| **Physical functioning** |  |  |  |  |  |
| Since caring for him*/*her, it seems like I’m tired all of the time. |  |  |  |  |  |
| My health has become worse since I’ve been caring for him*/*her. |  |  |  |  |  |
| **Self-efficacy regarding health care services** |  |  |  |  |  |
| I am confident I would know who to contact from the community services if I had a problem. |  |  |  |  |  |
| I feel I’d know where to get more information on any topic related to stroke or caring if I needed it. |  |  |  |  |  |
| **Caregiving value** |  |  |  |  |  |
| I really want to care for him*/*her. |  |  |  |  |  |
| Caring for him*/*her makes me feel good. |  |  |  |  |  |
| I enjoy caring for him*/*her. |  |  |  |  |  |
| **Family support** |  |  |  |  |  |
| It is very difficult to get help from the family in taking care of him*/*her. |  |  |  |  |  |
| The family works together at caring for him*/*her. |  |  |  |  |  |
| Since caring for him*/*her, I feel the family has abandoned me. |  |  |  |  |  |
| **Quality of the interactions with healthcare services** |  |  |  |  |  |
| I am satisfied with information I have been given about help available from the community services. |  |  |  |  |  |
| I am satisfied with help I’ve received from the community services when I’ve had a problem. |  |  |  |  |  |

| **Psychological functioning** | Not at all | Somewhat | Moderately | Suffi-ciently | Completely |
| --- | --- | --- | --- | --- | --- |
| I am able to accept my bodily appearance. |  |  |  |  |  |
|  | Very dissatisfied | Dissatisfied | Neither satisfied nor dissatisfied | Satisfied | Very satisfied |
| I am satisfied with myself. |  |  |  |  |  |
|  | Never | Sometimes | Often | Very often | Always |
| How often I have negative feelings such as blue mood, despair, anxiety, depression. |  |  |  |  |  |

| **Social capital** | Very dissatisfied | Dissatisfied | Neither satisfied nor dissatisfied | Satisfied | Very satisfied |
| --- | --- | --- | --- | --- | --- |
| How satisfied I am with my personal relationships. |  |  |  |  |  |
| How satisfied I am with my sex life. |  |  |  |  |  |
| How satisfied I am with the support I get from my friends. |  |  |  |  |  |

| **Security and material conditions** | Not at all | Somewhat | Moderately | A lot | Completely |
| --- | --- | --- | --- | --- | --- |
| How safe I feel in my daily life. |  |  |  |  |  |
| I have enough money to meet my needs. |  |  |  |  |  |
